# Potential cellular conformations of the CCN3(NOV) protein

**DOI:** 10.1186/1478-811X-2-9

**Published:** 2004-09-10

**Authors:** Stanimir Kyurkchiev, Herman Yeger, Anne - Marie Bleau, Bernard Perbal

**Affiliations:** 1 Laboratoire d' Oncologie Virale et Moléculaire, UFR de Biochimie, Université Paris 7-D. Diderot, Paris, France

**Keywords:** H295R adrenal and G59/540 glial tumor cell lines, CCN3, NOV, NOVH, nephroblastoma overexpressed protein, affinity purified antibodies to C-terminal domain, protein conformations, CCN proteins

## Abstract

**Aim:**

To study the cellular distribution of CCN3(NOV) and to determine if the carboxyterminus of CCN3 is hidden or masked due to high affinity interactions with other partners. CCN3 was detected using affinity purified antibodies (anti-K19M-AF) as well as a Protein A purified anti-K19M antibodies (anti-K19M IgG) against a C-terminal 19-aminoacid peptide (K19M) of human CCN3 protein. The antibodies were applied in indirect immunofluorescence tests and immunoenzyme assays on glial tumor cell line, G59, and its CCN3-transfected variant G59/540 and the adrenocortical cell line, NCI-H295R.

**Results:**

Anti-K19M-AF antibodies reacted against K19M peptide in ELISA and recognized two bands of 51 kDa and 30 kDa in H295R (adrenocortical carcinoma) cell culture supernatants by immunoblotting. H295R culture supernatants which contained CCN3 as shown by immunoblotting did not react with anti-CCN3 antibodies in liquid phase. Anti-CCN3 antibodies stained the surface membranes of non-permeabilized H295R and cytoplasm in permeabilized H295R cells. Similarly, anti-CCN3 stained surface membranes of G59/540, but did not react with G59 cells. Prominent cytoplasmic staining was observed in G59/540, as well as the cell footprints of G59/540 and H295R were strongly labeled.

**Conclusions:**

The K19M-AF antibody directed against the C-terminal 19-aminoacid peptide of CCN3 recognized the secreted protein under denaturing conditions. However, the C-terminal motif of secreted CCN3 was not accessible to K19M-AF in liquid phase. These anti-CCN3 antibodies stained CCN3 protein which was localized to cytoplasmic stores, cell membranes and extracellular matrix. This would suggest that cytoplasmic and cell membrane bound CCN3 has an exposed C-terminus while secreted CCN3 has a sequestered C-terminus which could be due to interaction with other proteins or itself (dimerization). Thus the K19M-AF antibodies revealed at least two conformational states of the native CCN3 protein.

## Introduction

The CCN3 protein belongs to an emerging family of growth regulators referred under the CCN acronym (cysteine-rich protein, Cyr61, connective tissue growth factor, CTGF, and the nephroblastoma overexpressed gene, nov; CCN 1–3 respectively) [1-3]. The CCN family now comprises six identified members with properties of both positive and negative regulators of cell growth, sharing a common multimodular organization. New members of the CNN family have been described over the past few years, and recent reviews on the CCN proteins highlight their intimate involvement in a variety of key biological processes including development, angiogenesis, and cancer [1-4].

The CCN3 (NOV) gene had been initially characterized as an integration site for the myeloblastosis associated virus MAV [5] which induces kidney tumors resembling nephroblastoma and Wilms tumor [6]. In human and animal tumors, the expression of the CCN3 gene was found to be altered either positively or negatively [7-11]. Experiments performed in our laboratory have established that CCN3 is a marker of tumor differentiation in Wilms tumors [12] and several other tumor types [unpublished observations]. Furthermore, an increasing amount of results assigns growth inhibitory functions to CCN3 in several conditions ([7,8,13-15], Manara et al. submitted).

The CCN proteins share a strikingly conserved multimodular organization with distinctive functional features [1]. From the amino to the carboxy terminus of these proteins, four modules can be recognized : an insulin-like growth factor (IGF) binding protein (IGFBP)-type motif, followed by a Von Willebrand type C (VWC) domain likely responsible for oligomerisation, a thrombospondin type 1 (TSP1) repeat, responsible for interaction with extracellular matrix proteins, and a carboxy-terminal module (CT), postulated to represent a dimerization domain, as it contains a cysteine-knot motif that is present and involved in the dimerization of several growth factors such as nerve growth factor (NGF), transforming growth factor -2 (TGF-2) and platelet derived growth factor BB (PDGFB).

The multimodular structure of CCN3 and other CNN proteins raises interesting questions as to participation of each individual module in conferring the biological properties to the full length proteins. Either the biochemical functions of the individual IGFBP, VWC, TSP and CT modules are indeed conserved and in sum determine the ultimate function of the full length protein, or each module confers on the whole protein specific biological functions which may vary from the conserved function, and either substitute or add to those of individual modules.

Application of the yeast two-hybrid system and co-precipitation strategies to identify proteins interacting with CCN3 has revealed that full length CCN3 interacts with several receptors, signaling molecules, and proteins of the extracellular matrix (16–19), suggesting functional involvement of CCN3 in cell signaling and adhesion regulation.

Our results also established that truncated isoforms of CCN3 could bind specific targets and pointed the CT domain of CCN3 as a critical determinant for protein interaction. This led us to hypothesize that truncated isoforms of CCN3 could also modulate its biological activity (3). The question therefore arises whether different conformational states exist due to multiple protein interactions and thereby the presentation of known antigenic epitopes.

In the present study we have used an immunological approach to establish the cellular distribution of CCN3 in cell lines representing adrenocortical and glioblastoma tumors and to ask whether the CT module of CCN3 exists in different conformational states depending on its involvement in protein interactions and cellular location. We now provide evidence that the CT end of CCN3 exists in more than one conformational state.

## Results

**Figure 1:**
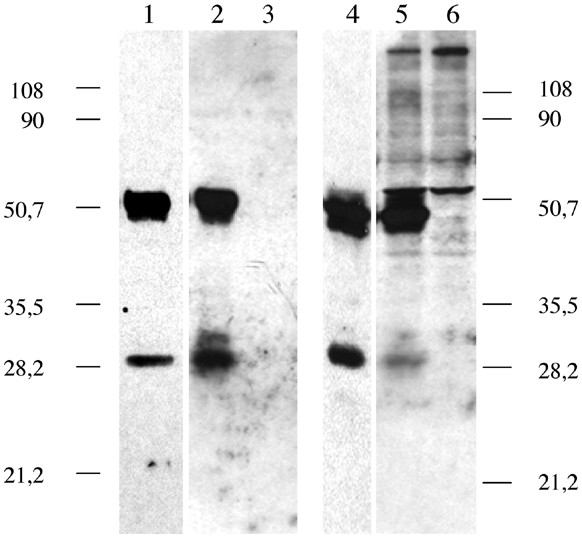
Western blot analysis. Representative gels illustrating the expression of CCN3 proteins in H295R, G59/540, and G59 cell lines. Conditioned medium and cellular lysates were electrophoresed under denaturing conditions and immunoblotted with anti-K19M IgG antibody. A. Starts: 1). supernatant collected form H295R cell line; 2). G59/540 transfected cells supernatant; 3). supernatant from G59 CCN3 negative cells; 4). lysate from H295R cells; 5). lysate from G59/540 cells; 6). lysate from G59 cells showing two bands at 48 kDa and 30 kDa.

Cell culture supernatants and cellular lysates from the H295R, G59/540, and parental G59 cell lines were electrophoresed under denaturing conditions and immunoblotted with anti-K19M IgG antibody. Immunoblot analysis revealed secreted forms of CCN3 for the H295R and G59/540 cell lines, consisting of two distinct bands at 51 kDa and 30 kDa [Figure 1A]. The latter likely corresponded to the previously described amino-truncated CCN3 isoform [3]. Intracellular CCN3 proteins were also detected in these cell lines. However, in addition to the two bands at 48 kDa and 30 kDa, two other high molecular species reacting positively with the antibodies were also detected in the lysates [Figure 1B]. The different sizes of these various isoforms likely results from post-translational modifications and oligomerisation of CCN3 protein.

**Figure 2:**
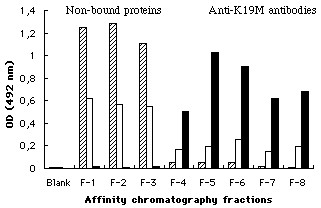
Reactivity of affinity purified anti-K19M-AF antibodies in ELISA. Affinity chromatography fractions were tested in wells coated with: a) GST 1 μg/ml (hatched columns); b) GST-CCN3 1 μg/ml (blank columns); c) K19M peptide 1 μg/ml (black columns). Column fractions F1 to F3 contain the flow through unbound proteins while fractions F4 to F8 contain antibody bound to K19M peptide that eluted with glycine buffer pH 2.8.

**Figure 3:**
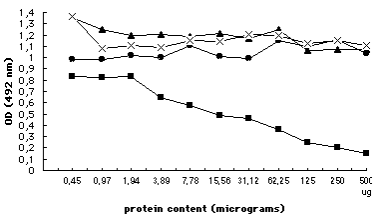
Absorption ELISA to check the binding of anti-K19M-AF to CCN3 in liquid phase. A fixed dilution of anti-K19M-AF (1/250 in 1% BSA) was mixed with a) serial dilutions of K19M peptide -- --- --- -- b) serial dilutions of H295R supernatant, positive for CCN-3 --- --- ---; c). G59 supernatant, negative for CCN-3 ---**x**----**x**---; d). 1% BSA in PBS pH 7.4 --- --- --- -- and applied to wells coated with K19M peptide. Each point repsent the average of 4 determinations.

When tested in ELISA, pre-incubation of the anti-K19M-AF antibodies with CCN3-containing H259R supernatant did not affect the binding of anti-K19M-AF antibodies to the K19M peptide coated on microtitre plates (Figure 2). Under identical conditions, the absorption of K19M-AF antibodies with serial dilutions of K19M peptide showed a dose-dependent absorption pattern with 7.78 μg/ml K19M peptide, yielding a 50% reduction in the binding of anti-K19M-AF to K19M peptide coated on plates (Figure 3). These results suggested that the K19M-AF antibodies did not recognize the CCN3 protein in its native configuration, whereas it can be detected in the same sample after denaturation and Western blotting.

**Figure 4:**
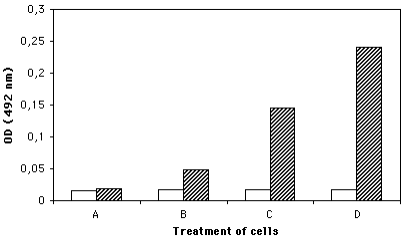
Detection of CCN3 expression on cultured cell lines by cell-ELISA. Cells from H295R line or glioblastoma G59 cells were treated with 1% bovine serum albumin in PBS, pH 7.4 (blank columns) or K19M-AF diluted 1/500 in 1%BSA (hatched columns). Treatment of cells: A – G59 non-permeabilized cells; B – C59 permeabilized cells; C – H295R non-permeabilized cells; D – H295R permeabilized cells. Each point repsent the average of 4 determinations.

When fixed and non-permebialized cells were used in cell-ELISA with anti-K19M-AF antibodies it was shown that positive reaction of the antibodies could be recorded with H295R cells which are known to synthesize and secrete CCN3 protein, while the reaction with G59 cells was in the ranges of the negative background. After permebialization of the cells the intensity of the reaction was increased but a significantly positive reaction was recorded with H295R cells (Figure 4).

Since CCN3 was secreted by H259R cells, it was important to check whether it could bind cell surface. Evidence for this would lend support to the previous suggestion of an autocrine mechanism of control by the CCN3 protein. To explore this possibility cells from H259R, G59/540 and G59 cell lines were incubated for 1 h on ice in the presence of supernatant containing CCN3 protein and then analyzed by cell ELISA as described above. The results obtained showed that such a treatment did not increase the intensity of the reaction with anti-K19M-AF bound to the cell surface. These experiments demonstrated that H295R, and G59/540 which expressed CCN3 on the cell surface and no further absorption occurred, and the control G59/540 cells did not absorb CCN3 from the culture supernatant (data not shown).

### Cellular Localization of CCN3

**Figure 5:**
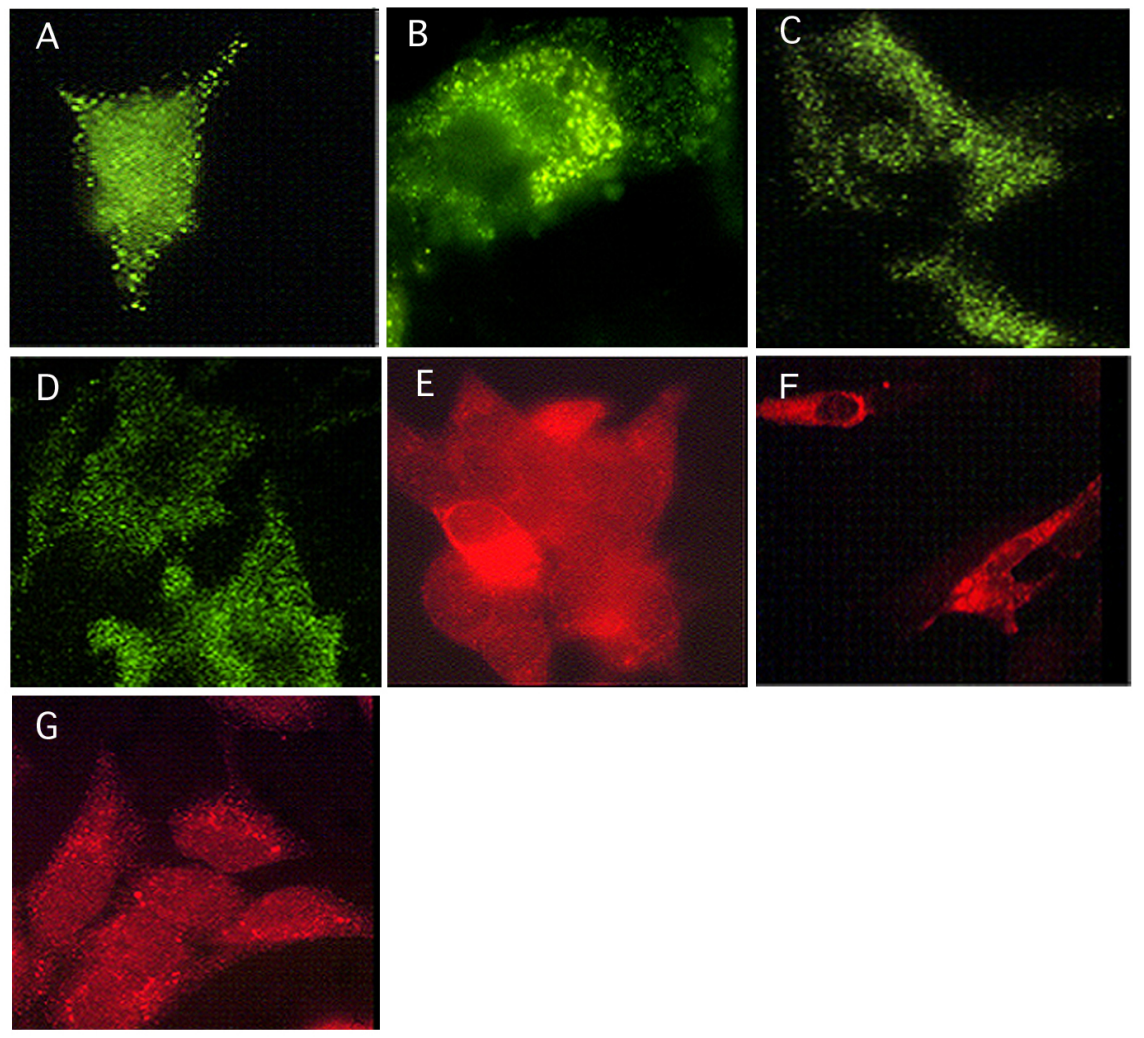
Indirect immunofluorescence staining with the anti-K19M IgG antibody. A, B The H295R and G540 cell lines, paraformaldehyde fixed and non-permeabilized, show a similar granular like surface membrane labeling pattern; C, D, Both in H295R and G540 cultures, paraformadehyde fixed and non-permeabilized, cell footprints are positively labeled; E, F, The H295R and G540 cell lines, formalin/ethanol fixed and permeabilized show a granular cytoplasmic labelling pattern that is perinuclear localized in ER and Golgi network; G, The G59/540 parent line, formalin/ethanol fixed and permeabilized, still shows a small amount of a perinuclear granular staining pattern.

Paraformaldehyde fixed, non-permeabilized H259R cells treated with the anti-K19M antibody (Protein A purified) exhibited immunofluorescent membrane specific staining distributed over the cell surface (Figure. 5A) while similarly treated G59 CCN3 negative cells did not stain (not shown). The CCN3-transfected glioblastoma cell line G59/540 stained positively with a similar localization of the reaction product (Figure 5B – G540). Interestingly, since cells grown on coverslips and fixed in paraformadehyde tend to slough, we did note the presence of positively staining cell footprints, suggesting deposition of CCN3 protein in a secretable extracellular matrix (Figures 5C,5D). After ethanol/formalin fixation and further permeabilization of the cells with 0.1% Triton X-100 the anti-K19M antibody (Protein A purified) gave an intensive granular fluorescence pattern which appeared perinuclear in a significant fraction of the cells with a similar pattern observed in H295R and G59/540 cells (Figure 5E,5F). On the other hand, the parent G59 cell line showed a weak, but still perinuclear cytoplasmic staining (Figure 5G). The latter may represent a smaller endogenous isoform of CCN3.

In summary, the results produced using an immunological approach would suggest multiple conformations of the C-terminal end which harbors the immunogenic epitopes. Furthermore, these variations are associated with cell bound and secreted forms of CCN3. Cytoplasmic localization indicated abundant CCN3 protein localized with ER and Golgi networks.

## Discussion

In this study we exploited the range of binding affinities present in polyclonal antibodies raised against the C-terminal peptide of CCN3 and analyzed with different immunoaffinity methods to ask whether CCN3 exists in alternate conformational forms in cell cultures and supernatants. Whereas immunocytochemistry of fixed, permeabilized and non-permeabilized cells yielded evidence of both cell surface membrane and cytoplasmic expression and topographical distribution of native CCN3, ELISA method and western immunoblot revealed different possible conformational forms of CCN3. Taken together the results of the assays suggest that native CCN3 assumes different configurations that either expose or sequester the C-terminal peptide depending on whether CCN3 is cell associated or free within the culture supernatant. When considered in the light of recent evidence indicating that CCN3 can associate with specific integrins at the cell membrane [23,24] the question also arises whether CCN3 associates with specific protein partners in the circulation and in the extracellular matrix produced by different cell types.

In these studies we focused on two cell lines, H295R and G59, representing adrenocortical and glial tissues, significantly different in their anatomical location and microenvironment. Since CCN3 has been demonstrated in plasma [25] it is conceptually feasible that CCN3 may be secreted by well organized ectodermal, mesodermal and endodermal cell types where it is expressed [26-28], and then is transported through complex extracellular matrix to enter the circulation. Moreover, as CCN3 has been shown to be expressed by endothelium [23,29], the source of the circulating CCN3 may be restricted to endothelium. Keeping to this scenario, tissue expression of CCN3 would be restricted to regional cell types and its arena of activity relegated to the extracellular matrix and resident cells. Interestingly, we could show *in vitro *that CCN3 is sequestered in cell footprints representing a secreted extracellular matrix. Cell footprints can be deposited by various cell types in different arrangements and consists of extracellular matrix, including a variety of basement membrane proteins [30-32]. In our studies we noted a more uniform and punctuate deposition of CCN3 in the footprints suggesting possible association with regularly arranged clustered partners (e.g. integrins), yet to be determined. Localization of CCN3 to footprints is perhaps expected since it has been shown to mediate adhesion of endothelial cells [23], in turn triggering intracellular phosphorylation signaling events.

This then raises the notion that proximity of CCN3 to cell surfaces could allow CCN3 to function in possible autocrine and paracrine mechanisms. Depending on the associating proteins, CCN3 would likely undergo specific conformational changes with potentially different functional outcomes. A variety of functional states may exist since CCN3 is expressed in secretable and non-secretable isoforms and contains motifs that overlap with other proteins and therein, additional binding partners [1,4]. Thus far the actual molecular function of native CCN3 has not been determined, although biologically it shows evidence of being able to regulate mitogenesis and motogenesis [3]. In turn, CCN3, compared to other CCN proteins, may be differentially regulated by mechanical stress [29]. As a heparan binding protein, CCN3 could associate with a large group of molecules at the cell membrane and in the extracellular matrix. Yeast two hybrid studies have indicated associations with fibulin 1C [16]. Other studies have shown CCN3 involved in calcium signaling [19,33], associated with Notch signaling [17], and able to trigger membrane mediated phosphorylation events [34] Some of the cellular effects of CCN3 may also be mediated by different isoforms operating at the level of the nucleus [35].

The biological significance of different conformations of CCN3 is not known. However, examples from other studies have suggested that conformational changes can occur in serum proteins due to binding of bivalent cations [36]. Since we recently reported that CCN3 may interact with Ca2+ binding proteins like fibulin-1C, modulate calcium uptake, and considering that Ca2+ binding modulates function of other protein partners such as integrins [reviewed in [33]], it is conceivable that secreted CCN3 could assume an altered conformation by binding bivalent cations, directly or indirectly, like Ca2+ present in culture media. Whether calcium or some other bivalent cation could be involved and how this could occur is still speculative as the sequence of CCN3 does not suggest any obvious cation binding properties. It is also possible that secreted CCN3 complexes with an as yet unknown partner thus sequestering the antigenic epitopes.

Altered expression of CCN3 in a variety of cancers may reflect maintenance of a normal homeostatic function of the cell of origin, or may indicate requirement of specific CCN proteins for maintaining the undifferentiated tumor state. One such example where the two possibilities are not yet resolved is in Wilms tumor, where CCN3 is abundantly expressed during normal nephrogenesis and in tumors [12]. Interestingly, CCN3 was originally identified during MAV virus induction of nephroblastoma but is not a direct target of WT1, the Wilms tumor suppressor gene [34]. Thus far few mutations have been described for CCN family and none for CCN3. CCN proteins have however been associated with a variety of cancers where they can be markedly overexpressed [11,37-39]. It may be that CCN proteins are not directly involved in tumorigenesis (e.g., Wilms tumor) but rather play supporting roles or may act as a negative regulator on malignant behavior reflecting their roles as integrators of cell-cell and cell-matrix communications. Thus having antibodies that can recognize the different isoforms of CCN proteins with great specificity and in respect to specific epitopes within the domains would be invaluable for quantitation [40] and for dissecting their functions in communication signaling.

## Conclusions

Our preliminary investigations here have revealed possible physical and functional states of native CCN3 localizing to cytoplasmic, cell membrane and extracellular matrix. Further complexity is added since shorter and larger isoforms of CCN3 can be detected using western blotting. The origin of these short forms is still not fully understood. As there do not appear to be alternate transcripts [1] this suggests post-translational processing including, in addition to variant glycosylation, phosphorylation, specific proteolytic events and sites. The C-terminal antibody recognizes these forms. The use of antibodies to other motifs in CCN3 will permit us to track the cleaved N-terminal peptide which potentially could be functionally active as it resembles IGFBP [1]. Therefore the cleavage products of CCN3 in concert with native CCN3 may also be involved in several aspects of the regulation of growth factor activity at the cell membrane or its management in extracellular repositories.

Finally, cells can coordinately express a variety of CCN proteins that are closely related, for example CCN1-3 with cross-over and opposite functional effects yet bearing similar functional domains. Evidence is starting to surface that they might compete for binding partners, such as integrins, thus forming protein complexes with different biological consequences to cell behavior [18,19]. It will be important to understand how stoichiometric changes in CCN protein concentrations can change the behavior of cells, thus opening up opportunities for therapeutic manipulations in disease. It is obvious that there will be a necessity for antibody reagents and quantitative methodologies to enable these studies.

## Materials and methods

### Cell Lines

NCI H259R (American Type Cell Collection) is a human adenocortical carcinoma cell line and was cultured in DMEM/F12 supplemented with 2.5 % Nu-serum plus ITS+ supplement (Sigma Co, St. Louis, USA). H295R cells have been characterized and were shown to secrete high levels of CCN3 protein [20,21]. The glioblastoma cell line (G-59) has been described previously [22]. CCN3 expressing G59/540 sublines were obtained following transfection of G59 with pCMV CCN3 plasmid and G418 selection [13]. These cell lines and their derivatives were used in cell ELISA and indirect immunofluorescence labeling experiments as described below.

### Antibodies

Antibodies against C-terminal peptide K19M were used in these experiments after either purification by an antigen specific affinity chromatography (anti-K19M-AF) or by Protein A chromatography (anti-K19M IgGs).

### Antibody Affinity Purification and Characterization

The K19M C-terminal peptide (KNNEAFLQELELKTTRGKM) of human CCN3 protein was coupled to CNBr activated Sepharose 4B (Pharmacia Biotech, Uppsala, Sweden) following the protocol recommended by the manufacturer. Briefly, 3 mg of peptide were dissolved in 5 ml of 0.1 M NaCO_3 _pH 9.0 containing 0.5 M NaCl (coupling buffer) and added to 3.5 ml CNBr-Sepharose swelled gel and the mixture was rotated end-over-end for 2 hours at room temperature. Excess ligand was eluted with 20 ml of coupling buffer and the gel was incubated in 0.1 M Tris-HCl buffer pH 8.0 for 2 hours at room temperature. The gel was washed 5 times in cycles consisting of 20 ml of 0.1 M acetate buffer pH 4.0 followed by 20 ml of 0.1 M Tris-HCl buffer pH 8.0, each containing 0.5 M NaCl, and then packed into a PD-10 column.

The rabbit anti-K19M antiserum was absorbed with 1 mg/ml human serum albumin to remove cross-reactivity with human plasma and dialyzed overnight at 4°C against phosphate buffer pH 7.0. An aliquot of 3.5 ml serum was loaded on the affinity column and the flow through and the unbound proteins were collected in 3 ml fractions followed by thorough washing of the column with the loading buffer. The K19M peptide bound antibodies were eluted with 9 ml of 0.1 M Tris-glycine buffer pH 2.8 in 3 ml fractions that were collected into test tubes containing 100 μl 1 M Tris buffer pH 8.0. All column fractions were tested by ELISA for the presence of antibodies reacting against K19M peptide and the positively reacting fractions were further purified by affinity chromatography on pre-packed HiTrap Protein A columns (Pharmacia Biotech, Uppsala, Sweden) as recommended. Affinity purified antibody preparations were further tested to determine their reactivities and specificities. The affinity purified anti-K19M-AF antibodies reacted against the K19M peptide when tested in serial dilutions in ELISA (see below). The titers of K19M-AF antibodies were comparatively lower as compared to the unfractionated K19M antiserum. This finding was not unexpected as it likely reflects the polyclonal composition of the primary rabbit antiserum and differences in the content of the specific monoclonal specificities in the antiserum. Importantly, the affinity purified antibodies recognized the K19M peptide when coated on a solid phase.

### K19M Peptide Enzyme-linked Immunoabsorbent Assay (ELISA)

Affinity purified antibodies were titered by ELISA. Individual wells of polystyrene 96-well flat bottom plates (NUNC) were coated with 1 μg/ml of K19M peptide diluted in coating buffer (0.05 M carbonate buffer pH 9.6) by incubation overnight at 4°C. The unsaturated protein binding sites were blocked with 300 μl/well 2% BSA for 1 h at room temperature. The primary anti-K19M antiserum and affinity purified antibodies were added in serial dilutions in duplicates and the wells were incubated for 2 h at room temperature. After thorough washing the wells were incubated with goat anti-rabbit IgG serum conjugated with peroxidase (Sigma Co) diluted 1/5000 in blocking buffer for 1 h at room temperature. The bound enzyme activity was revealed by adding the enzyme substrate 0.5 mg/ml ortho-phenylenediamine in citrate buffer pH 5.0 containing 0.5 μl/ml H_2_0_2_. The enzyme reaction was stopped by addition of 50 μl/well of H_2_SO_4 _and the color reaction was read at 492 nm in a MicroELISA reader.

### Cell Enzyme-Linked Immunoabsorbent Assay (Cell ELISA)

Tumor cell lines were cultured in complete medium in 96-well flat bottom plates (Corning) to form a subconfluent monolayer and further incubated overnight in serum free medium. The cells were washed 3 times for 5 min each with phosphate buffered saline (PBS, pH 7.2) and cells were fixed by treatment with ice-cold methanol for 30 min warming to room temperature. The endogenous peroxidase activity was blocked with 3% H_2_O_2 _in distilled water for 7 min at room temperature followed by 3 × 5 min washes in PBS, pH 7.2. Non-specific binding was blocked with 1% bovine serum albumin (BSA) for 1 h at room temperature. Cells were washed once in PBS and incubated with K19M-AF diluted in 1%BSA for 2 h at room temperature. After 3 × 10 min washes in PBS goat-anti rabbit IgG conjugated with peroxidase diluted 1/10000 in 1% BSA-PBS was added for 1 hour at room temperature. The cells were washed again in PBS and the bound enzyme activity was developed by adding ortho-phenylendiamine (5 mg/10 ml citrate buffer, pH 5.0) containing 5 μl of 30% H_2_O_2_. The color reaction was stopped by adding 50 μl of 10% H_2_SO_4 _and the intensity was read at 492 nm in a MicroELISA reader.

### Gel Electrophoresis and Western blotting

To prepare proteins for immunoblotting, cells were lysed in NP40 buffer (50 mM Tris hydrochloride, pH 8.0, 150 mM NaCl, 5 mM EDTA and 2% NP40) with protease inhibitors (Cocktail Tablets, complete, Roche) and phosphatase inhibitors (50 mM NaF, 2 mM sodium orthovanadate) for 30 min at 4°C. After centrifugation at 15 000 g, extracts were stored at -80°C until use. CCN3 proteins in the conditioned medium were concentrated on Heparin Sepharose (Amersham, Uppsala, Sweden) as described by Chevalier et al (1998). Briefly, supernatants were incubated overnight with heparine and then washed 4 times in PBS containing protease inhibitors. Bound CCN3 was dissociated using 2-mercaptoethanol in Laemmli buffer, boiled for 10 min and then centrifuged to keep the free protein. Heparin Sepharose concentrated samples and cellular extracts were subjected to electrophoresis under reducing conditions in 12.5% polyacrylamide gels. Separated proteins were subsequently transferred to nitrocellulose by a semi-dry blotter (LKB Biotech, Sweden) as recommended by the supplier. The nitrocellulose sheet was blocked by incubation for 1 hour at room temperature with 5% nonfat milk in PBST (PBS with 0.2% Tween 20, pH 7.4). The membrane was then incubated in the same buffer with the anti-K19M IgG (1/2000) and then washed extensively. The blots were further incubated in goat anti-rabbit IgG conjugated with peroxidase (1/10 000 in blocking solution, Sigma Co, USA) for 1 hour at room temperature. Revelation was performed using the chemoluminescence protocol and reagents (Pierce, Rockford, IL, USA).

### Indirect Immunofluorescence Labeling

Cells were grown on alcohol flamed coverslips, rinsed in PBS and fixed in cold 70% ethanol containing 10% formalin (Sigma) for 10 min on ice, and stored in PBS. Immunofluorescence labeling was performed at room temperature. For this procedure, coverslips were placed into weighing boats [Sigma; 4.5cm × 4.5cm] maintaining cell side up. Cells were further permeabilized in 0.5% Triton X-100 in PBS for 15 min and then blocked in 5%FBS/PBS for 30 min.

Anti-K19M IgGs antibodies were applied at 1:1000 dilution in 5%FBS/PBS for 1 hour with intermittent rotation, followed by 5 washes in PBS containing 0.1% Tween 20. Subsequently, the cells were incubated in anti-rabbit IgG serum conjugated with either Alexa 488 (green fluorescence) or Alexa 594 (red fluorescence), in 5%PBS/BSA for 1 hour. After final washes in PBS/Tween 20 followed by one wash in PBS cells were mounted with antifade mounting medium (Bio-Rad, France), excess liquid adsorbed with filter paper, and coverslips sealed with clear nail polish. Immunofluorescence images were captured on 400 ASA film and processed further with Adobe Photoshop (version 7.0).

## List of abbreviations

None.

## Competing interests

None declared.

## Authors' contributions

SK carried the affinity chromatography and immunoenzyme experiments;

HY carried out immunofluorescence labeling experiments;

AMB carried out IgG purification and Western blots;

BP conceived the study design and coordinated and edited the manuscript
